# High *CRLF2* expression associates with *IKZF1* dysfunction in adult acute lymphoblastic leukemia without *CRLF2* rearrangement

**DOI:** 10.18632/oncotarget.10437

**Published:** 2016-07-06

**Authors:** Zheng Ge, Yan Gu, Gang Zhao, Jianyong Li, Baoan Chen, Qi Han, Xing Guo, Juan Liu, Hui Li, Michael D. Yu, Justin Olson, Sadie Steffens, Kimberly J. Payne, Chunhua Song, Sinisa Dovat

**Affiliations:** ^1^ Department of Hematology, Zhongda Hospital, Medical School of Southeast University, Nanjing, 210009, China; ^2^ Department of Hematology, The First Affiliated Hospital of Nanjing Medical University, Jiangsu Province Hospital, Nanjing, 210029, China; ^3^ Department of Pediatrics, Pennsylvania State University Medical College, Hershey, PA, 17033, USA; ^4^ Sydney Kimmel Medical College at Thomas Jefferson University, Philadelphia, PA, 19107 USA; ^5^ University of Wisconsin at Stout, Menomonie, WI, 54751, USA; ^6^ Loma Linda University, Department of Pathology and Human Anatomy, Loma Linda, CA, 92350, USA

**Keywords:** CRLF2, IKZF1, adult, acute lymphoblastic leukemia

## Abstract

Overexpression of cytokine receptor-like factor 2 (*CRLF2*) due to chromosomal rearrangement has been observed in acute lymphoblastic leukemia (ALL) and reported to contribute to oncogenesis and unfavorable outcome in ALL. We studied B-ALL and T-ALL patients without *CRLF2* rearrangement and observed that *CRLF2* is significantly increased in a subset of these patients. Our study shows that high *CRLF2*expression correlates with high-risk ALL markers, as well as poor survival. We found that the *IKZF1*-encoded protein, Ikaros, directly binds to the *CRLF2* promoter and regulates *CRLF2* expression in leukemia cells. CK2 inhibitor, which can increase Ikaros activity, significantly increases Ikaros binding in ALL cells and suppresses *CRLF2* expression in an Ikaros-dependent manner. *CRLF2* expression is significantly higher in patients with *IKZF1* deletion as compared to patients without *IKZF1* deletion. Treatment with CK2 inhibitor also results in an increase in *IKZF1* binding to the *CRLF2* promoter and suppression of *CRLF2* expression in primary ALL cells. We further observed that CK2 inhibitor induces increased H3K9me^3^ histone modifications in the *CRLF2* promoter in ALL cell lines and primary cells. Taken together, our results demonstrate that high expression of *CRLF2* correlates with high-risk ALL and short survival in patients without *CRLF2* rearrangement. Our results are the first to demonstrate that the *IKZF1*-encoded Ikaros protein directly suppresses *CRLF2* expression through enrichment of H3K9me^3^ in its promoter region. Our data also suggest that high *CRLF2* expression works with the *IKZF1* deletion to drive oncogenesis of ALL and has significance in an integrated prognostic model for adult high-risk ALL.

## INTRODUCTION

Cytokine receptor-like factor 2 (*CRLF2*), also known as thymic stromal lymphopoietin (TSLP) receptor, is a type I cytokine receptor. The *CRLF2* subunit generates the functional receptor for TSLP by forming a heterodimeric complex with interleukin-7 receptor alpha (IL7Rα) [1]. Both the cytokine and its receptor have been implicated in tumorigenesis [2]. Overexpression of *CRLF2* in leukemia has been reported [3–6] and was correlated with poor outcome in pediatric acute lymphoblastic leukemia (ALL) [2, 7, 8]. However, the correlation of *CRLF2* expression with the prognosis of adult ALL patients has not been fully determined.

The mechanisms of *CRLF2* overexpression in leukemia are not fully understood [2]. Several studies have described the correlation between elevated CRLF2 mRNA expression and genomic lesions affecting CRLF2 [8]. *CRLF2* rearrangements (*IGH–CRLF2;* PAR1 deletion and *P2RY8–CRLF2*) have been reported to result in elevated expression of wild-type CRLF2 [3–5, 7]. Overexpression of *CRLF2* occurs in 15% adult and pediatric ALL [9, 10]. However, the *CRLF2* rearrangement subtype is reported to account for only about half of ALL with high *CRLF2* expression [11, 12]. These findings suggest that factors other than CRLF2 rearrangement can be responsible for CRLF2 overexpression. *IKZF1* deletion occurs in 43% of pediatric ALL with *CRLF2* overexpression [11], although it is present in ~80% of Ph-like ALL with CRLF2 rearrangement [13]. *IKZF1* deletions and *CRLF2* overexpression were also identified in 12% and 11%, respectively, of Japanese Ph-like ALL patients, [7]. However, it is unknown whether *CRLF2* is a direct target of IKZF1 or if IKZF1 deletion is associated with increased *CRLF2* expression in ALL, particularly in cases without *CRLF2* rearrangement.

Recently, we reported the IKZF1 global binding profiling in ALL cells and found that IKZF1 regulates the expression of its targets through chromatin remodeling in ALL [14–17]. Our ChIP-seq data showed IKZF1 binding peaks in the promoter region of *CRLF2*. We also found that CK2 inhibitors could increase the tumor suppressor activity of IKZF1 and act as a functional activator of the IKZF1 protein [14–16]. However, it is still unclear whether and how IKZF1 regulates *CRLF2* expression.

Here we examined *CRLF2* expression in adult ALL patients without *CRLF2* rearrangement, and analyzed the correlation of *CRLF2* expression with prognosis in adult ALL patients. We observed high expression of *CRLF2* in high-risk leukemia and this correlates with the poor clinical outcome. We also identified that *CRLF2* as a direct target of IKZF1. IKZF1 binds to and suppresses *CRLF2* expression in ALL leukemic cells. The CK2 inhibitor, TBB, which restores IKZF1 tumor suppressor activity, increased the suppression of *CRLF2* expression and *IKZF1* knockdown blocks the TBB-induced changes. Deletion of *IKZF1* is significantly associated with *CRLF2* overexpression in adult ALL. We also found that *IKZF1* suppresses *CRLF2* expression by chromatin remodeling through recruitment of H3K9me^3^ to the promoter. Our findings indicate that *IKZF1* deletion may be responsible for the overexpression of *CRLF2* in high-risk ALL without CRLF2 rearrangement. High expression of *CRLF2* may work in conjunction with *IKZF1* deletion to drive oncogenesis of ALL and both of them have significance in an integrated prognostic model for adult high-risk ALL.

## RESULTS

### High CRLF2 expression is associated with poor prognosis in adult ALL without CRLF2 rearrangement

**Figure 1:**
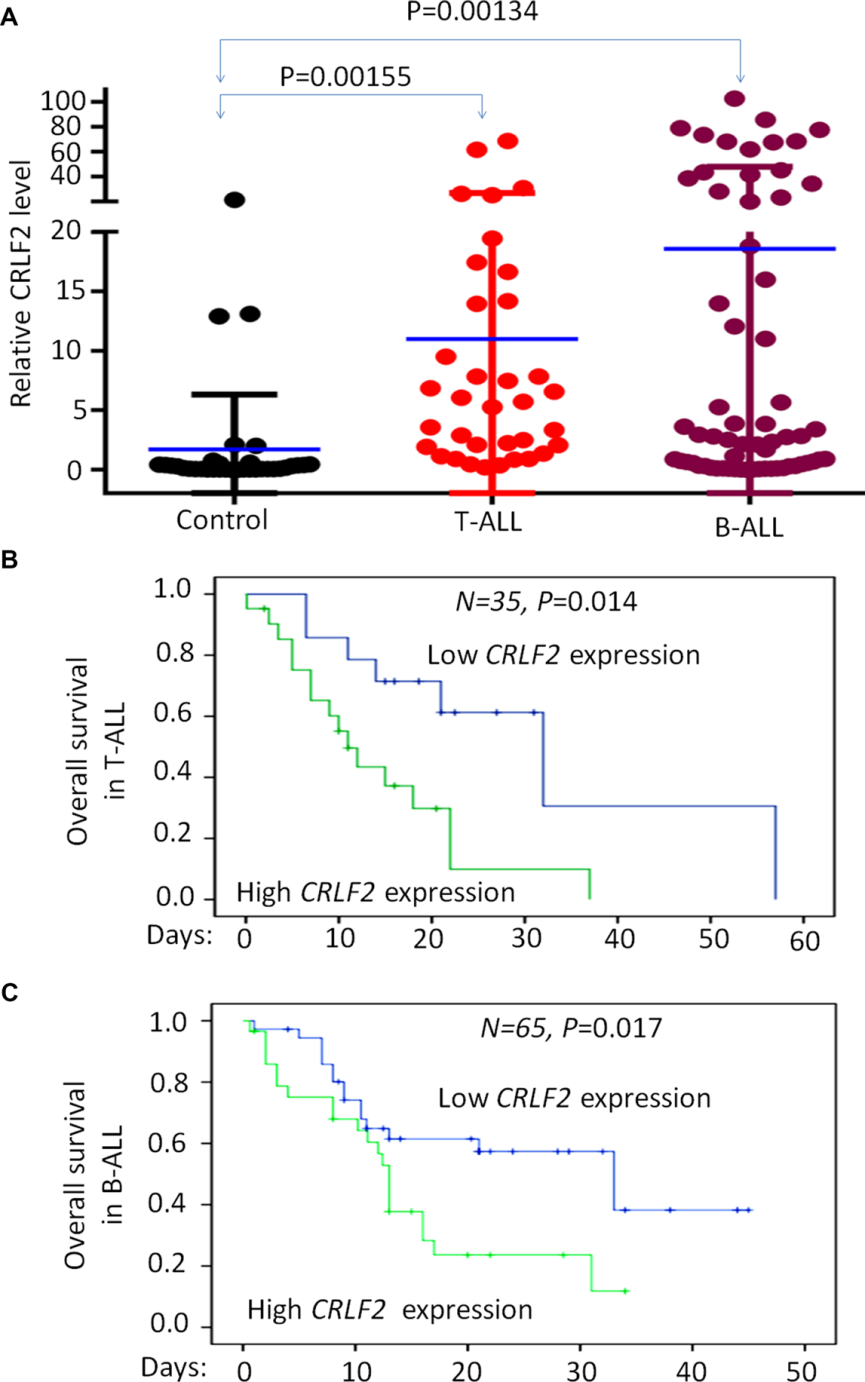
Correlation of *CRLF2* overexpression and correlation with survival in adult ALL (**A**) Comparison of *CRLF2* mRNA level in B-ALL and T-ALL with normal BM control by qPCR. (**B–C**) *CRLF2* expression with overall survival in adult T-ALL (B) and B-ALL (C).

CRLF2 is highly expressed in the reported microarray cohort study of ALL patients (Supplementary Figure 1). Here we examined *CRLF2* mRNA expression in 100 newly diagnosed adult ALL patients without *CRLF2* rearrangement (B-ALL 65 cases and T-ALL 35 cases). We found that the *CRLF2* mRNA level is significantly higher in both B-ALL and T-ALL patients when compared to that of normal bone marrow controls (Figure 1A).

We divided the patients into high *CRLF2* expression (Quartile 1-Quartile 2) or low expression (Quartile 3-Quartile 4) groups and analyzed the relationship between *CRLF2* expression and clinical characteristics in the cohort of B-ALL and T-ALL respectively (Supplementary Table S1 and S2). High *CRLF2* expression in B-ALL patients showed a significant association with higher median WBC (45.8 × 10^9^/L vs. 19.4 × 10^9^/L, *P* = 0.035) and a higher percentage of WBC ≥ 30 × 10^9^/L (85.7% vs. 38.9%, *P* < 0.0001), which are markers of poor prognosis in B-ALL (Supplementary Table 1). In addition, patients with high *CRLF2* expression were more frequently positive for the stem cell marker CD34 (85.7% vs. 55.6%, *P* = 0.010) and the myeloid markers CD13 (60.7% vs. 33.3%, *P* = 0.029) and CD33 (59.3% vs. 30.6%, *P* = 0.023), which are indicative of a poor prognosis in B-ALL patients. We also observed that the high *CRLF2* expression group had a higher frequency of splenomegaly (58.6% vs. 27.8%, *P* = 0.012), indicating extramedullary infiltration. Importantly, detection of Ikaros isoform6 (IK6), produced by the most common type of *IKZF1* deletion, was statistically higher in the B-ALL patients with high *CRLF2* expression (31.0% vs. 8.3%, *P* = 0.019)(Supplementary Table 1).

The T-ALL patients with high *CRLF2* expression exhibited a trend toward higher median WBC (82.0 × 10^9^/L vs. 24.8 × 10^9^/L, *P* = 0.256) than those with low *CRLF2* expression. Patients with high *CRLF2* expression had lower hemoglobin (114.0 g/L vs. 144.0 g/L, *P* = 0.039), as well as higher percentages of blasts in peripheral blood (82.0 vs. 33.0, *P* = 0.032), higher rates of positivity for CD34 (71.4% vs. 21.4%, *P* = 0.006), and CD33 (61.9% vs. 21.4%, *P* = 0.036) and higher frequencies of lymph node infiltration (90.5% vs. 57.1%, *P* = 0.039) as compared to patients with low *CRLF2* expression (Supplementary Table 2). No significant differences in *CRLF2* expression were observed with respect to age, gender, platelet, bone marrow blasts or complex karyotype (Supplementary Tables 1 and 2). These data indicate that a high level of *CRLF2* expression is associated with poor prognostic markers in this cohort of the adult ALL patients without *CRLF2* rearrangements.

We further analyzed the overall survival (OS) in the cohort of B-ALL and T-ALL respectively. We found that cases with high *CRLF2* expression showed significantly shorter OS than cases with low *CRLF2* expression in both B-ALL (13.0 months vs. 33.0 months, *P* = 0.017) and T-ALL (11 months vs. 32.0 months, *P* = 0.014) (Figure 1B and 1C). These data further indicate that high *CRLF2* expression is associated with poor outcome in ALL patients without *CRLF2* rearrangements.

### IKZF1 binds to the promoter of CRLF2 and regulates its expression in ALL

**Figure 2:**
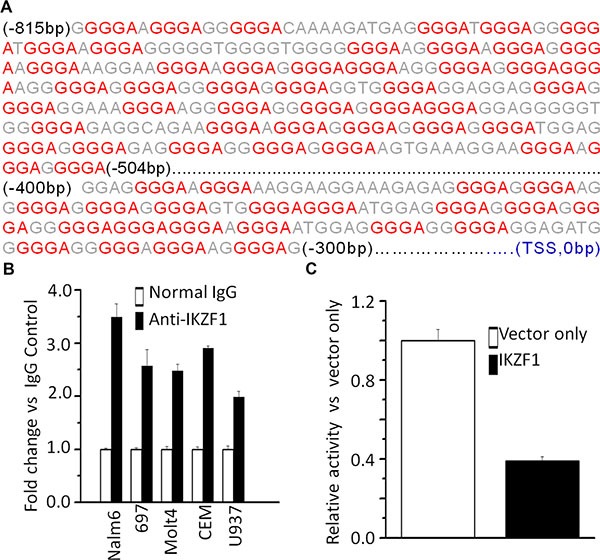
IKZF1 binds the promoters of *CRLF2* (**A**) CRLF2 promoter region with conserved IKZF1 binding motif (red); (**B**) qChIP data for IKZF1 binding on *CRLF2* promoter in leukemic cells; (**C**) The promoter activity of *CRLF2* promoters measured by luciferase reporter assay following transfection with *IKZF1* or control vector in HEK293 cells.

**Figure 3:**
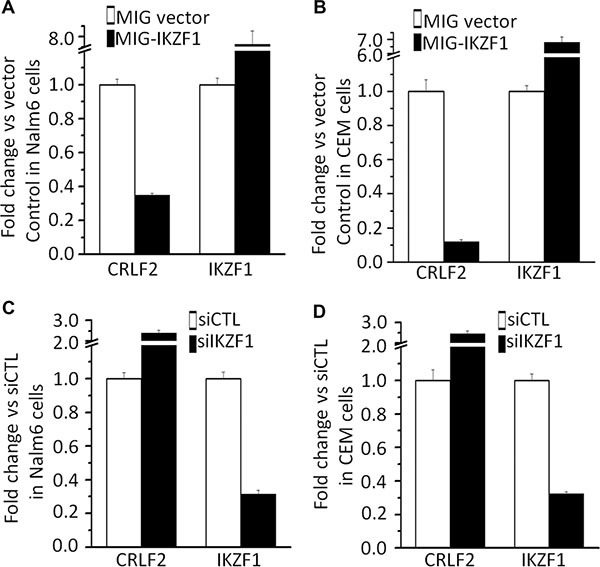
IKZF1 suppresses *CRLF2* expression (**A–B**) Expression of *CRLF2* and *IKZF1* in Nalm6 cells (A) and CEM T-ALL cells (B) transduced with vector containing *IKZF1* as compared to control vector. (**C–D**) qPCR of *CRLF2* and *IKZF1* expression in Nalm6 cells (C) and CEM cells (D), following *IKZF1* shRNA treatment as compared to scramble shRNA cells. Gene expression is determined by RT-qPCR using total RNA isolated from the cells transfected with scramble shRNA (siControl) or *IKZF1* shRNA (si*IKZF1*) for 2 days.

**Figure 4:**
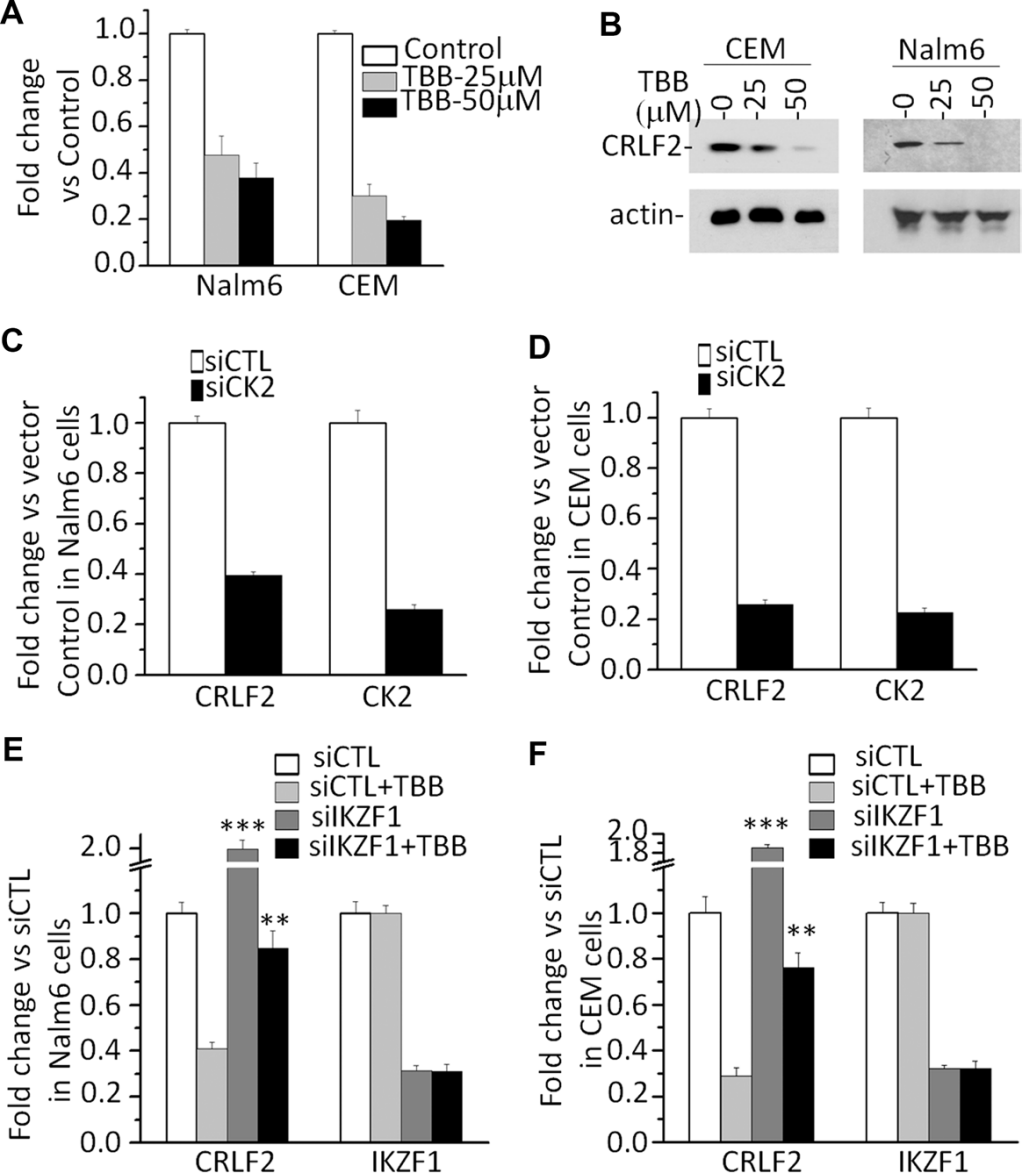
CK2 inhibitor-TBB suppress the expression of *CRLF2* in an IKZF1-dependent manner (**A–B**) Effect of CK2 inhibitor-TBB) which functions as Ikaros activator, on expression of CRLF2 mRNA level (A) and protein level (B) in Nalm6 and CEM cells with TBB treatment for 2 days. (**C–D**) Effect of CK2 knockdown on the expression of *CRLF2* in Nalm6 (C) and CEM (D) cells. (**E–F**) IKZF1 knockdown rescues the TBB-induced change of *CRLF2* in Nalm6 (E) and CEM (F) cells. Cells was treated with 25 μM TBB for 2 days.

To further address the potential link between high *CRLF2* expression with high-risk markers and poor outcome in adult ALL without *CRLF2* rearrangement, we analyzed transcription factor motifs in the promoter region of *CRLF2*. As we expected, many core Ikaros binding motifs (GGGA) were identified in the promoter region of *CRLF2* (Figure 2A). Notably, our ChIP-seq data showed strong binding peaks for *IKZF1* in the *CRLF2* promoter region [14, 15]. qChIP, assays showed significant binding of *IKZF1* at the *CRLF2* promoter region in both B-ALL (Nalm6 and 697) and T-ALL (CEM and MOLT-4) cells, but very weak binding in U-937 AML cells (Figure 2B). In addition, we observed that *IKZF1* suppresses the promoter activity of *CRLF2* by luciferase reporter assay (Figure 2C). These data indicate a direct effect of *IKZF1* on transcription of *CRLF2*. Moreover, we found that expression of *IKZF1* suppresses *CRLF2* mRNA levels in both Nalm6 (Figure 3A) and CEM cells (Figure 3B). Conversely, efficient *IKZF1* knockdown increased *CRLF2* expression in Nalm6 (Figure 3C) and CEM cells (Figure 3D). Furthermore, treatment of Nalm6 and CEM cells with CK2 inhibitor (TBB) could suppress *CRLF2* mRNA and protein levels in a dose-dependent manner (Figure 4A and 4B). CK2 knockdown with shRNA also induced the suppression of *CRLF2* expression in Nalm6 (Figure 4C) and CEM (Figure 4D) cells. Importantly, *IKZF1* knockdown with shRNA could block the TBB-induced decrease of *CRLF2* expression in Nalm6 (Figure 4E) and CEM (Figure 4F) cells. These data indicate that *CRLF2* is the direct target of *IKZF1* and that *IKZF1* suppresses *CRLF2* expression in ALL.

### Correlation of IKZF1 deletion with high high CRLF2 expression in primary ALL

**Figure 5:**
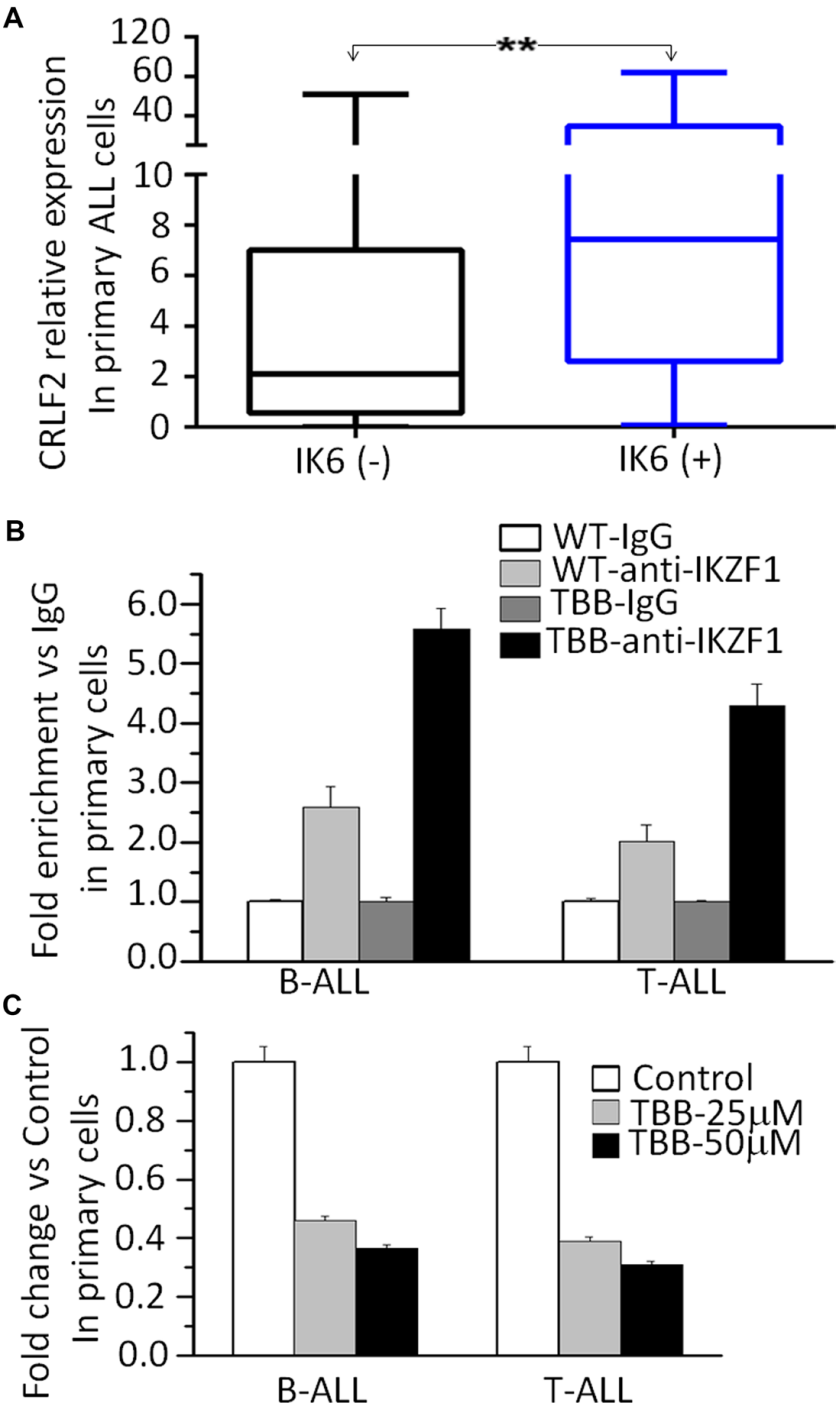
IKZF1 binds to the *CRLF2* promoters and *IKZF1* deletion results in changes of its expression in primary ALL cells (**A**) Comparison of *CRLF2 expression* in patients with or without IKZF1 deletion, presented as *CRLF2*/*GAPDH*. The detection method for Ik6 (the most common Ikaros deletion) was done as our previously reported (23). (**B**) CK2 inhibitor-TBB increased the IKZF1 binding to the promoters of *CRLF2* in primary B-ALL(left) and T-ALL (right) cells. (**C**) Effect of CK2 inhibitor-TBB functioning as IKZF1 activator on expression of *CRLF2* in primary B-ALL(left) and T-ALL (right) cells with TBB treatment for 2 days.**P < 0.01.

To provide further supporting that that *CRLF2* is a direct target of IKZF1 and that IKZF1 suppresses *CRLF2* expression, we analyzed the relationship between *IKZF1* deletion and high *CRLF2* expression in primary ALL. We found a higher incidence of IK6, the most common protein produced by *IKZF1* deletion in B-ALL patients in the high *CRLF2* expression group (31% vs 8.3%, *P* = 0.019) (Supplementary Table 1). Moreover, we found that *CRLF2* expression was significantly higher in patients with *IKZF1* deletion than in patients without *IKZF1* deletion (Figure 5A). These data suggest that *IKZF1* deletion may contribute to high *CRLF2* expression in patients. Furthermore, treatment of primary ALL cells with the CK2 inhibitor, TBB, induced an increase in IKZF1 binding at the *CRLF2* promoter region in both primary B-ALL and T-ALL as compared to untreated controls (Figure 5B). Assay by qPCR also showed that TBB treatment inhibited expression of *CRLF2* mRNA in a dose-dependent manner (Figure 5C). These results indicate that IKZF1 binds to the promoter of *CRLF2* and that treatment with CK2 inhibitors, which can enhance IKZF1 tumor suppressor activity, resulted in suppression of *CRLF2* expression in primary cells.

### Enhanced Ikaros activity due to CK2 inhibition increases H3K9me3 at the CRLF2 promoter

**Figure 6:**
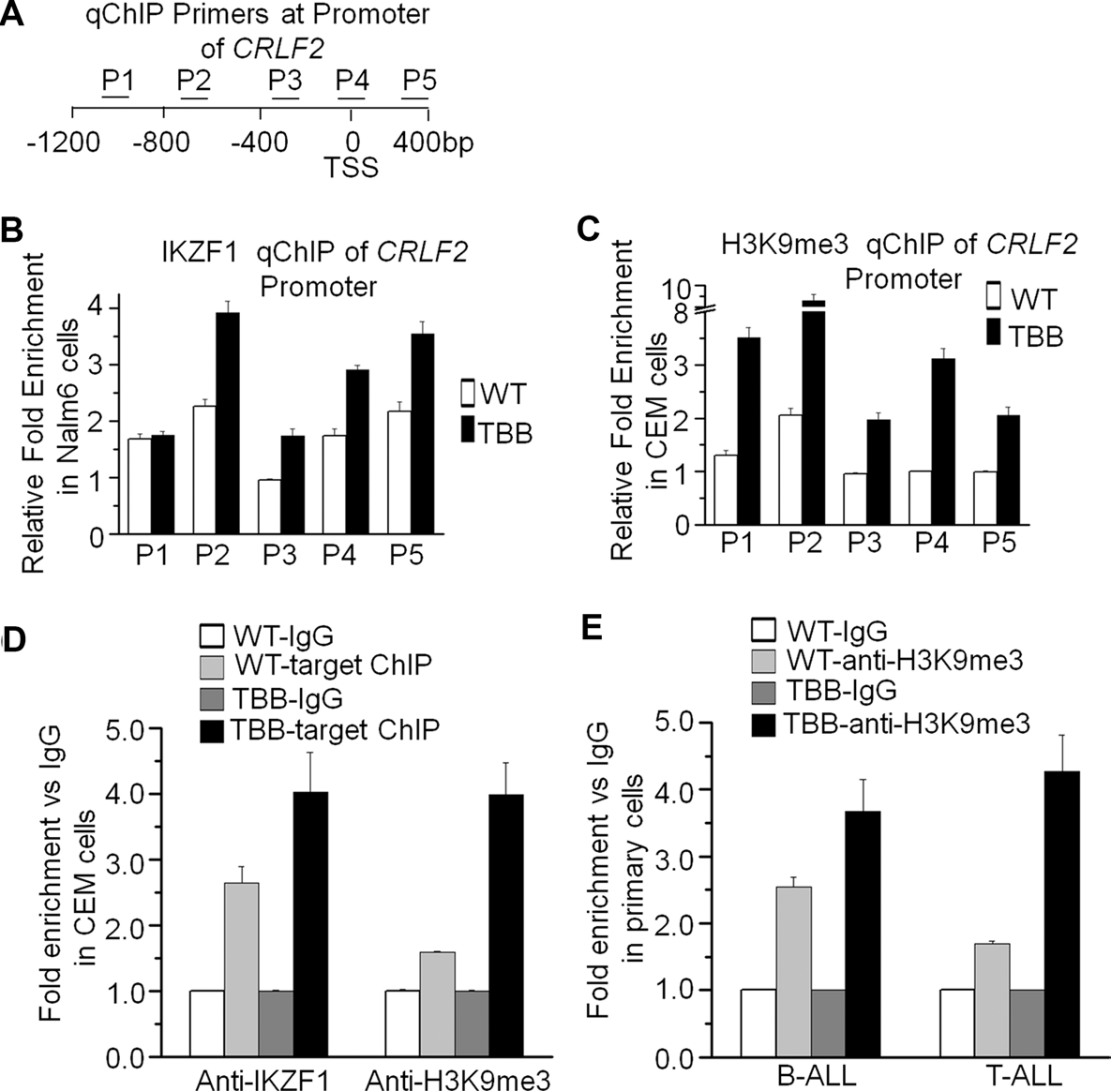
CK2 inhibitor-TBB by restoring Ikaros function induces transcriptionally repressive epigenetic changes at the *CRLF2* promoter (**A**) Schematic depiction of the qChIP primers used to analyze epigenetic changes following TBB treatment. Positions of primers used for qChIP are indicated relative to the *CRLF2* transcription start site (TSS). (**B–C**) The qChIP analysis of the distribution of *IKZF1* (B) and H3K9me3 (C) at the *CRLF2* promoter in Nalm6 cells cultured with or without 50 μM TBB for 2 days. (**D**) The qChIP data of *IKZF1* and H3K9me3 enrichment at the *CRLF2* promoter in CEM T-ALL cells cultured with or without TBB. (**E**) The qChIP data of H3K9me3 enrichment at the *CRLF2* promoter in primary ALL cells cultured with or without TBB. Graphed data are presented as mean +/− SEM of triplicates of three independent experiments.

Our previous publications have reported that IKZF1 regulates gene expression through chromatin remodeling [5, 18]. To further explore the epigenetic mechanism by which IKZF1 regulates *CRLF2*, we performed ChIP assay and amplified the resulting *CRLF2* promoter sequences with five pairs of primers as shown in Figure 6A. The results showed that TBB, which enhances IKZF1 function, significantly increases binding of IKZF1 at the CRLF2 promoter as compared with that in untreated Nalm6 B-ALL cells (WT) (Figure 6B). Moreover, TBB also clearly enhances the binding of H3K9me^3^ (a hallmark of heterochromatin) at the *CRLF2* promoter region in Nalm6 cells (Figure 6C). Similar results were observed in the CEM T-ALL cell line (Figure 6D). However, we did not find enrichment of other histone modification markers in the *CRLF2* promoter region (data not shown). Further, we found that TBB treatment increases both the binding of IKZF1 (Figure 5B) and the presence of the H3K9me^3^ histone modification (Figure 6E) at the *CRLF2* promoter in primary ALL cells. These data indicated that enhanced IKZF1 activity suppresses *CRLF2* expression via increased H3K9me^3^ in its promoter region.

## DISCUSSION

**Figure 7:**
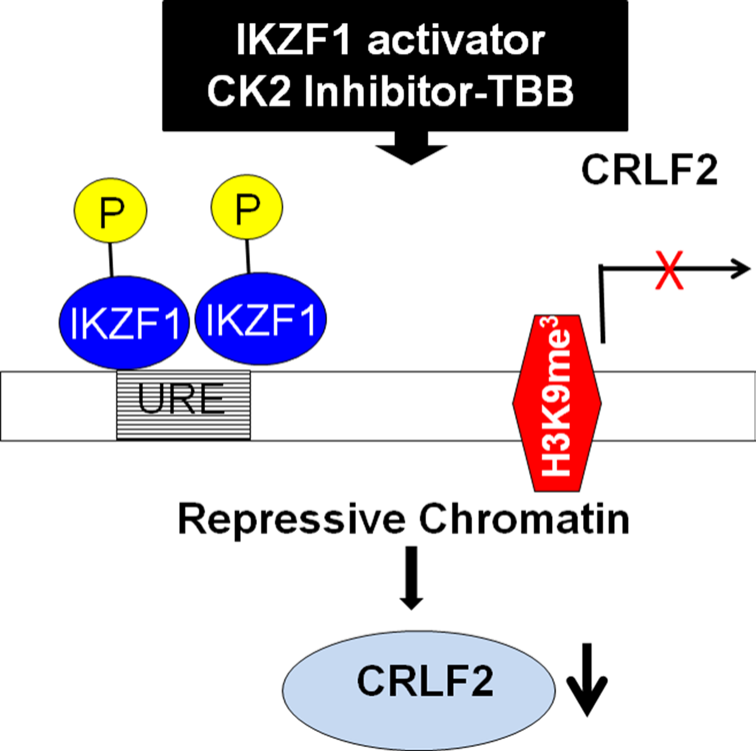
Proposed model of IKZF1 activator inducing suppression of *CRLF2* expression

Genetic alterations at the *CRLF2* locus are associated with overexpression of CRLF2 in ALL [15–18]. CRLF2 rearrangement occurs at high frequency in Down Syndrome ALL [3, 9, 19] and in patients of Hispanic/Latino ethnicity [21]. However the rate of CRLF2 overexpression occurs at a higher frequency in patients than CRLF2 rearrangement [9, 22]. Thus *CRLF2* rearrangement is unable fully to explain the mechanisms underlying CRLF2 overexpression in ALL patients. Here we provide the first report that *CRLF2* is a direct target of IKZF1 and that IKZF1 regulates CRLF2 expression via induction of the H3K9me^3^ histone modification at its promoter (Model see Figure 7). We also show that Ikaros deletion is associated with high CRLF2 which suggests another mechanism, in addition to genetic alteration of the *CRLF2* locus, is responsible for CRLF2 overexpression in ALL.

In this cohort study, we focus on ALL patients without *CRLF2* rearrangement. We found that high expression of CRLF2 is correlated with unfavorable prognostic factors in the patients. Strikingly, the overall survival was significantly shorter in both T-ALL and B-ALL patients with elevated CRLF2. These observations suggested that CRLF2 overexpression is also correlated with high-risk adult ALL even when the *CRLF2* locus has not undergone rearrangement.

It is worth noting that we observed expression of Ik6 in 18.5% of B-ALL without CRLF2 rearrangement and particularly that 75.0% of these IK6+ patients also showed overexpressed *CRLF2*. These data indicate that *IKZF*1 deletion with *CRLF2 over* expression but no CRLF2 rearrangement may distinguish a subgroup of adult ALL patients with unfavorable prognosis. This data also revealed the prognostic correlations for *IKZF1* deletion and high *CRLF2* expression in ALL patients. More importantly, we observed that *CRLF2* expression is significantly higher in patients with Ik6, suggesting a role for the *IKZF1* deletion in the elevated *CRLF2* expression.

Our previous publications have demonstrated that IKZF1 exerts anti-tumor effects by regulating the expression of its target genes such as c-Myc, PI3KCD, KDM5B, etc. [14–17]. Here we observed that IKZF1 binds to the promoter region of *CRLF2* and directly suppresses the promoter activity of *CRLF2*. Our data provide evidence that IKZF1 suppresses *CRLF2* expression and conversely, that *IKZF1* knockdown increases *CRLF2* expression in ALL cells. We also showed that treatment with the CK2 inhibitor, TBB, which can restore/enhance Ikaros function, could increase IKZF1 binding at the *CRLF2* promoter and suppress *CRLF2* expression in both ALL cell lines and primary cells in an IKZF1 dependent manner. These data indicate that *CRLF2* is a direct target of IKZF1 and that increasing IKZF1 activity increases the suppression of *CRLF2* expression in ALL.

We previously reported that IKZF1 regulates gene expression through chromatin remodeling [15, 19]. Here, we observed that increasing IKZF1 activity via the CK2 inhibitor, TBB, increases H3K9me^3^, a suppressive chromatin marker, at the *CRLF2* promoter. This indicates that suppression of *CRLF2* expression via chromatin remodeling is also a mechanism underlying IKZF1 anti-oncogenesis in ALL, particularly high-risk ALL.

In summary, we report that *CRLF2* is highly expressed and significantly correlated with poor clinical outcome in a cohort of adult ALL patients without *CRLF2* rearrangements. We found that Ikaros deletion (characterized by Ik6 expression) is significantly correlated with high *CRLF2* expression. We are the first to show that *CRLF2* is a direct target of IKZF1, that IKZF1 regulates *CRLF2* expression, and that restoration of IKZF1 activity using the CK2 inhibitor, TBB, can suppress *CRLF2* expression via chromatin remodeling in ALL. Our results strongly implicate *CRLF2* overexpression in conjunction with *IKZF1* deletion in the oncogenesis of ALL and suggest that these should be integrated in future prognostic models for adult ALL patients.

## MATERIALS AND METHODS

### Patients and samples

Bone marrow (BM) samples from 100 patients with newly diagnosed ALL and without *CRLF2* rearrangements (65 B-ALL and 35 T-ALL) were collected between June 2008 and June 2015 at the First Affiliated Hospital of Nanjing Medical University. Thirty normal bone marrow samples were obtained from the volunteer. Written informed consent was provided before enrollment in the study by all patients and volunteers in accordance with the Declaration of Helsinki. The study was approved by the Ethics Committee of The First Affiliated Hospital of Nanjing Medical University, Jiangsu Province Hospital, Nanjing, China. The diagnosis of ALL were made according to the morphologic, immunophenotypic, cytogenetic and molecular criteria of WHO Diagnosis and Classification of ALL (2008).

### Cytogenetic and molecular analyses

Cytogenetic analysis, determination of Ik6 [23], *CRLF2* rearrangements [3, 10, 21], *BCR-ABL* fusion gene/Ph chromosome [8, 23] were performed as previously described. qPCR was performed with qSTAR SYBR Master Mix (OriGene) on StepOne Plus Real-time PCR system (Applied Bioscience). In order to quantitate gene expression value in patients' samples, template standards and primers against Homo sapiens gene *CRLF2* (OriGene, USA) and Homo sapiens housekeeping gene *GAPDH* (OriGene, USA) were obtained from OriGeneTechnologies (Rockville, MD, USA). Gene expression values of the genes of interest (GOI) were achieved in each patient by a formula obtained with a scatter graph of the Ct values from the serial dilutions of template standard following manufacturer's instruction and as previously reported [16, 24]. The expression level of the GOI was subsequently normalized to the housekeeping gene, expressed as gene expression value of GOI/GAPDH. Each experiment was performed in triplicate. All patients were placed in high or low *CRLF2* expression groups (Quartile 1-Quartile 2 vs. Quartile 3-Quartile 4) and the cut-off values were determined by SPSS 17.0 [16].

The qPCR for *CRLF2* expression was similarly performed as above in Nalm6 and CEM cells. The results were normalized to those obtained with 18sRNA and presented as fold induction over vector controls. Primers: *18s RNA*, Sense:5′-GTAACCCGTTGAACCCCATT-3′, Antisense: 5′- CCATCCAATCGGTAGTAGCG-3′;*CRLF2* Sense: 5′- GCCAGACCCGAAATCCATCT −3′, Anti-sense: 5′- CCTGGAAGTTCCCTTGGTGTAT −3′.

### Cell culture reagents, plasmid construction, and retroviral gene transfer

The Nalm6 [25] and 697 [11] have been previously described. CCRF-CEM (CEM), MOLT-4 and U-937 cells were obtained from the American Type Culture Collection (ATCC, Manassas, VA). Cell lines were cultured in RPMI 1640 medium (Cellgro, USA) supplemented with 10% fetal bovine serum (Hyclone, USA). HEK 293T cells were cultured in DMEM (Cellgro) supplemented with 10% fetal calf serum and 1% L-glutamine (Cellgro). Cells were incubated at 37°C in a humidified atmosphere of 5% CO_2_. Primary human B-ALL and T-ALL cells were cultured in RPMI 1640 medium (Cellgro) supplemented with 10% fetal bovine serum (Hyclone). 4, 5, 6, 7-Tetrabromobenzotriazole (TBB) was purchased from Sigma (St. Louis). Cells were cultured with or without TBB and collected for total RNA isolation. Human HA-tagged *IKZF1* retroviral construct and retroviral production was described previously [14, 16, 26, 27].

### Luciferase assay

The promoter of *CRLF2* (−1000 bp to +200 bp) was cloned into pGL4.15 vector (Promega, WI, USA). The transient luciferase assay was performed in HEK293T cells using the Promega luciferase assay reagents and measured with luminometer following the manufacture's instruction [14, 16]. The firefly luciferase activities were calculated as fold change relative to values obtained from pGL4.15 vector only control cells, and expressed as a percentage of pcDNA3. 1-*IKZF1* transfection-induced luciferase activity versus that of pcDNA3.1 vector. All transfection and reporter assays were performed independently, in triplicate, at least three times.

### Quantitative chromatin immunoprecipitation (qChIP)

The qChIP assays were performed by incubation of the chromatin with antibodies against IKZF1 [14–16], H3K9me3 (Abcam, USA) or normal rabbit IgG (Abcam) as a control [14–16, 23]. Enrichment of the ChIP sample over input was evaluated by qPCR with three or more replicates, using specific primers in the promoter region of *CRLF2* as shown in Supplementary Table 3 (forward: 5′-AGGGGA AGGGAGGGGAAGGGAAAG-3′, reverse: 5′-CCCTTTCCTCCCCTCCCCTCCT-3′). Relative concen- tration of the qPCR product was presented as the fold change of the level of DNA-*IKZF1* and DNA-H3K9me3 samples in comparison to controls.

### *IKZF1* shRNA knockdown

The Nalm6 cells were transiently transfected with human *IKZF1* shRNA constructs in the GFP vector (pGFP-v-RS) (Origene) using the Neon Transfection System (Invitrogen, USA). We used ascrambled 29-mer shRNA cassette in the pGFP-v-RS vector as a control. Knockdown of *IKZF1* was confirmed by measurement of *IKZF1* mRNA level with qPCR [14, 16, 27]. Primers: IKZF1-F: 5′-GGCGCGGTGCTCCTCCT-3′, IKZF1-R: 5′-TCCGACACGCCCTACGACA-3′.

### Western blot

Cell lysate from cells was prepared as previous reported [28]; Twenty microgram protein was boiled in SDS sample buffer for 10 minutes; the resulting samples were run on SDS-PAGE and transferred to the membrane. Membranes were blocked with 5% nonfat dry milk at room temperature for 1 h and then incubated overnight at 4°C with primary antibody (anti-CRLF2, 1:500; Santa Cruz). After being washed, the membrane was incubated with goat anti-rabbit IgG conjugated to horseradish peroxidase (1:3000) at room temperature for 2 h. The blots were developed by the enhanced chemiluminescence technique (ECL Plus, Amersham, Arlington Heights, IL) according to the manufacturer's instructions.

### Statistical analysis

We divided patients into high or low *CRLF2* expression groups (Q1-Q2 vs. Q3-Q4) [16, 24]. Overall median differences between the two groups were evaluated using a Mann–Whitney *U*-test. Overall frequency differences in the two groups were analyzed using a χ^2^ test. Survival analysis was calculated using the Kaplan–Meier method. All statistical analyses were performed using the SPSS version 17.0, and *P* < 0.05 was considered statistically significant.

The experimental data were shown as the mean value with bars representing the standard error of the mean (SEM). Determinations of statistical significance were performed using a Student *t*-test for comparisons of two groups or using analysis of variance (ANOVA) for comparing multiple groups. *P* < 0.05 was considered statistically significant.

## SUPPLEMENTARY MATERIALS FIGURE AND TABLES


