# Supplementary material for: High CRLF2 expression associates with IKZF1 dysfunction in adult acute lymphoblastic leukemia without CRLF2 rearrangement
Source: Oncotarget. 2016 Jul 6;7(31):49722–32. doi: 10.18632/oncotarget.10437 (PMC5226542; doi:10.18632/oncotarget.10437)
Supplement: Supplementary file 1 [file oncotarget-07-49722-s001.pdf]

High *CRLF2* expression associates with *IKZF1* dysfunction in adult acute lymphoblastic leukemia without *CRLF2* rearrangement

Supplementary Materials

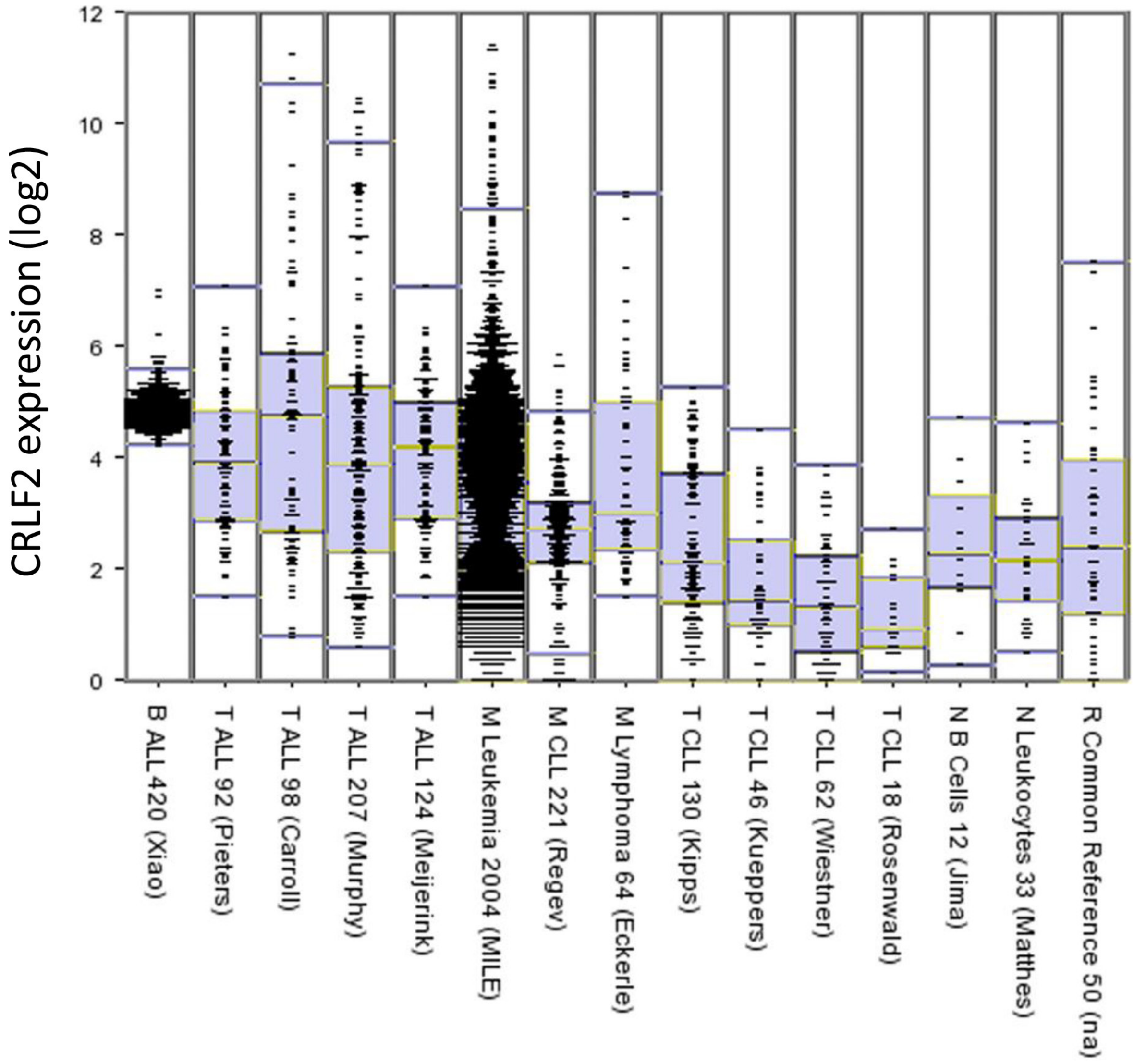

Supplementary Figure S1: CRLF2 high expression in ALL cohort study. The microarray data for CRLF2 (208303_s_at) expression generated with MegaSampler (*n* = 3581, MAS5.0) from human oncogenomics sever crossing all microarray database, which includes B-ALL, T-ALL, Mixed ALL (M ALL), T-CLL, and normal control (N B cells, N leukocytes and common reference) cohort studies.

Supplementary Table S1: Correlation of CRLF2 expression with clinical features in adult B-ALL

Characteristics	High CRLF2 expression (*N* = 29)	Low CRLF2 expression (*N*=36)	*P* value
Age (years)			
median (range)	38.5(14.0–77.0)	27.0(14.0–70.0)	0.418
Sex (%)			
male	48.3	52.8	0.718
WBC, $\times 10^9$/L			
median (range)	45.8(3.0–488.0)	19.4(0.9–398.4)	0.035
$\geq 30 \times 10^9$/L	85.7	38.9	0.000
HGB, g/L			
median (range)	89.0(48.0–150.0)	90.0(44.0–157.0)	0.765
PLT, ×109/L			
median (range)	47.0(8.0–269.0)	35.0(4.0–292.0)	0.529
LDH (u/L)			
median (range)	592.0(214.0–3257.0)	753.0(175.0–7142.0)	0.516
Blasts (%) median range			
bone marrow	90.4(66.0–97.0)	88.8(47.0–100.0)	0.533
peripheral blood	80.5(34.0–97.0)	70.0(0.0–100.0)	0.051
Immune phenotype,%			
CD34+	85.7	55.6	0.010
CD13+	60.7	33.3	0.029
CD33+	59.3	30.6	0.023
Extramedullary infiltration (%)			
liver	24.1	8.8	0.189
spleen	58.6	27.8	0.012
lymph node	41.4	47.1	0.651
Genetics (%)			
IKZF1 deletion (IK6)	31.0	8.3	0.019
BCR/ABL1 fusion gene(Ph+)	53.8	34.3	0.127
complex karyotype	15.8	21.7	0.925

Supplementary Table S2: Correlation of CRLF2 expression with clinical features in adult T-ALL

Characteristics	High CRLF2 expression ($N = 21$)	Low CRLF2 expression ($N = 14$)	P value
Age (years)			
median (range)	27.0(14.0–54.0)	32.5(14.0–49.0)	0.606
Sex (%)			
male	81.0	78.6	1.000
WBC, $\times 10^9$/L			
median (range)	82.0(3.0–546.0)	24.8(1.0–153.0)	0.256
$\geq 100 \times 10^9$/L	38.1	28.6	0.721
HGB, g/L			
median (range)	114.0(56.0–151.0)	144.0(90.0–171.0)	0.039
PLT, ×109/L			
median (range)	45.0(17.0–219.0)	86.0(22.0–223.0)	0.521
LDH (u/L)			
median (range)	755.0(131.0–4263.0)	1717.0(205.0–4905.0)	0.344
Blasts (%) median (range)			
bone marrow	80.2(24.0–98.8)	75.9(20.0–95.6)	0.548
peripheral blood	82.0(4.0–94.4)	33.0(1.0–84.0)	0.032
Immune phenotype (%)			
CD34+	71.4	21.4	0.006
CD13+	52.4	21.4	0.089
CD33+	61.9	21.4	0.036
Extramedullary infiltration (%)			
liver	28.6	0.0	0.061
spleen	57.1	28.6	0.166
lymph node	90.5	57.1	0.039
Genetics (%)			
NOTCH1 mutation	81.3	72.7	0.662
FBWB7 mutation	6.3	27.3	0.273
PTEN mutation	17.6	7.1	0.607
PHF6 mutation	28.6	25.0	1.000
DNM2 mutation	81.0	92.9	0.627
IL7R mutation	5.3	15.4	0.552
complex karyotype	20.0	14.3	1.000

Supplementary Table S3: CRLF2 primer for qChIP

CRLF2-P1-F: 5′-GTGAGCCAAGATCGTGC	CRLF2-P1-R: 5′-GAGCTGGAGTCTCACTC
CRLF2-p2-F: 5′-AGG GGA AGG GAG GGG AAG GGA AAG	CRLF2-P2-R: 5′-CCC TTT CCT CCC CTC CCC TCC T
CRLF2-P3-F: 5′-AAGTGAAAGGAAGGGAAGGGAGGGGA	CRLF2-P3-R: 5′-CCT TTC CCT TCC CCT CCC CTC C
CRLF2-P4-F: 5′-CACTGTGCTTCCTGCCTGTAAT	CRLF2-P4-R: 5′-AGAGGTCACCATCGATTTTAAAAAGT
CRLF2-P5-F: 5′-GCAAGGAGGAGCAGGTAATTATTT	CRLF2-P5-R: 5′-CAAAGCAAACCACAAACCAAAA
